# Fluoxetine ameliorates cartilage degradation in osteoarthritis by inhibiting Wnt/β-catenin signaling

**DOI:** 10.1371/journal.pone.0184388

**Published:** 2017-09-19

**Authors:** Kentaro Miyamoto, Bisei Ohkawara, Mikako Ito, Akio Masuda, Akihiro Hirakawa, Tadahiro Sakai, Hideki Hiraiwa, Takashi Hamada, Naoki Ishiguro, Kinji Ohno

**Affiliations:** 1 Division of Neurogenetics, Center for Neurological Diseases and Cancer, Nagoya University Graduate School of Medicine, Nagoya, Japan; 2 Department of Orthopaedic Surgery, Nagoya University Graduate School of Medicine, Nagoya, Japan; 3 Center for Advanced Medicine and Clinical Research, Nagoya University Hospital, Nagoya, Japan; The University of Hong Kong, HONG KONG

## Abstract

Abnormal activation of the Wnt/β-catenin signaling is implicated in the osteoarthritis (OA) pathology. We searched for a pre-approved drug that suppresses abnormally activated Wnt/β-catenin signaling and has a potency to reduce joint pathology in OA. We introduced the TOPFlash reporter plasmid into HCS-2/8 human chondrosarcoma cells to estimate the Wnt/β-catenin activity in the presence of 10 μM each compound in a panel of pre-approved drugs. We found that fluoxetine, an antidepressant in the class of selective serotonin reuptake inhibitors (SSRI), down-regulated Wnt/β-catenin signaling in human chondrosarcoma cells. Fluoxetine inhibited both Wnt3A- and LiCl-induced loss of proteoglycans in chondrogenically differentiated ATDC5 cells. Fluoxetine increased expression of *Sox9* (the chondrogenic master regulator), and decreased expressions of *Axin2* (a marker for Wnt/β-catenin signaling) and *Mmp13* (matrix metalloproteinase 13). Fluoxetine suppressed a LiCl-induced increase of total β-catenin and a LiCl-induced decrease of phosphorylated β-catenin in a dose-dependent manner. An *in vitro* protein-binding assay showed that fluoxetine enhanced binding of β-catenin with Axin1, which is a scaffold protein forming the degradation complex for β-catenin. Fluoxetine suppressed LiCl-induced β-catenin accumulation in human OA chondrocytes. Intraarticular injection of fluoxetine in a rat OA model ameliorated OA progression and suppressed β-catenin accumulation.

## Introduction

Osteoarthritis (OA) is a progressively degenerative joint disorder and causes chronic disability in elderly people and is one of the major health problems worldwide [1]. OA is characterized by degradation of extracellular matrix (ECM) molecules, loss of articular cartilage, and formation of osteophytes. No rational medical therapy is currently available for OA except for palliative pain control and physiotherapy, before the patient undergoes surgery. In healthy adult cartilage, chondrocytes are regulated by fine-tuned gene expressions of transcriptional factors, extracellular matrix (ECM) molecules, and their catabolic enzymes for maintenance of ECM [2]. In OA cartilage under mechanical stresses, ECM synthesis and ECM degradation are compromised in articular chondrocytes, as observed in hypertrophic chondrocytes in endochondral ossification [2]. Indeed, cartilage degeneration in human OA gradually worsens with gradual decreases of collagen type II, Sox9 (sex determining region Y-Box 9), and aggrecan, as well as gradual increases of collagen type X and Indian hedgehog, again as observed in hypertrophic chondrocytes during normal morphogenesis. A master transcription factor, Sox9, coordinates a postnatal genetic program for proper ECM homeostasis including production of abundant proteoglycans [3]. Meanwhile, matrix metalloproteinases (MMPs), aggrecanases (ADAMTS4 and 5), and other matrix proteases are expressed in chondrocytes to degrade ECM [4]. MMP13, one of the MMPs, efficiently degrades collagen type II [1]. Chondrocyte abnormalities in OA thus cause loss of chondrocytes, reduced elasticity of articular cartilage, and formation of aberrant osteophytes, which leads to loss of tolerance to mechanical stress of articular cartilage.

Wnt/β-catenin signaling regulates crucial aspects of determination of cell fate during adult tissue homeostasis by regulating expressions of target genes. Most Wnt ligands activate the canonical Wnt/β-catenin signaling, whereas some may not [5, 6]. In the absence of Wnt ligands involved in the canonical Wnt/β-catenin signaling, β-catenin is steadily phosphorylated by casein kinase 1 (CK1) and glycogen synthase kinase 3 (GSK3) in a degradation complex assembled by Axin1 and adenomatous polyposis coli (APC), and is subsequently degraded through the ubiquitin/proteasome pathway [7]. The Wnt ligands or lithium chloride (LiCl), a GSK3 inhibitor, suppresses phosphorylation of β-catenin, and subsequently suppresses degradation of β-catenin. Consequently, β-catenin is accumulated in the cytoplasm and is translocated into the nucleus to interact with T-cell factor/lymphoid enhancing factor (TCF/LEF) to activate transcription of the Wnt/β-catenin target genes. Wnt/β-catenin signaling is associated with chondrocyte differentiation and OA pathology [8–10]. Some genes encoding positive regulators and targets for Wnt/β-catenin signaling are up-regulated in the mouse OA cartilages and the human articular cartilage explants [11, 12]. Down regulation of MMPs by Wnt/β-catenin signaling in human OA chondrocytes [13] are predicted to be beneficial for OA. However, Wnt3A treatment induces abnormal de-differentiation in chondrocytes derived from adult human articular cartilage [14], and tissue-specific overexpression of β-catenin in articular chondrocytes leads to osteoarthritis-like phenotype [15]. Abnormal upregulation of genes encoding key molecules in Wnt/β-catenin signaling is observed in articular cartilage of rodent OA models [16, 17], in human OA-derived chondrocytes [18], and in the degraded areas of human articular cartilage [19]. These results are in accordance with a previous study that Wnt/β-catenin signaling induces degradation of proteoglycans in rat articular chondrocytes [20]. Indeed, Wnt3A induces rapid expression of the *Mmp13* gene in mouse primary chondrocytes [21]. High serum levels of FRZB (Frizzled related protein) and DKK1 (dickkopf 1 homolog), which are secreted antagonists for Wnts, are associated with the reduced risk of hip OA [22]. In addition, gene variants of *FRZB*, a secreted antagonist for Wnts, are risk factors for hip and knee OA [23, 24]. Indeed, conditional activation of β-catenin in articular chondrocytes in adult mice leads to premature chondrocyte differentiation and development of an OA-like phenotype [15, 25]. On the contrary, specific inhibition of β-catenin signaling in articular chondrocytes induces cell apoptosis and OA-like cartilage destruction in transgenic mice [26]. These studies indicate that finely tuned regulation of β-catenin is essential for normal functions of chondrocytes. Wnt/β-catenin signaling is one of the essential signaling pathways in cells, and inactivation of Wnt/β-catenin signaling may cause defects of cell proliferation and differentiation in multiple organs. A strong inhibitor of Wnt/β-catenin signaling is thus likely to be toxic, and may cause osteoporosis [27, 28]. In contrast, systemic upregulation of β-catenin may promote proliferation of tumors [7, 11, 19]. Thus, adverse effects of a small compound that modulates Wnt/β-catenin signaling should be carefully scrutinized.

Drug repositioning strategy, in which a drug already used for a specific disease is applied to another disease, has been gaining increasing attention from academia and industry [29, 30]. The advantage of this strategy is that the identified drugs can be readily applied to clinical practice, because the optimal doses, adverse effects, and contraindications are already established. A drug identified by the drug repositioning strategy may also have an extensive inhibitory effect on Wnt/β-catenin signaling, but we know that the unexpected effects are tolerated in humans without showing overt adverse effects.

The purpose of this study is to identify a clinically applicable drug, which inhibits Wnt/β-catenin signaling for OA. Using the drug repositioning strategy, we identified that fluoxetine, an antidepressant with a selective serotonin-reuptake inhibiting activity, suppressed activated Wnt/β-catenin signaling in chondrocytes. In chondrogenically differentiated ATDC5 cells, fluoxetine decreased activated Wnt/β-catenin signaling, suppressed degradation of proteoglycans, and increased expression of *Sox9*. We found that fluoxetine directly enhanced binding of β-catenin with Axin1 *in vitro*. Intraarticular injection of fluoxetine ameliorated cartilage damage in a rat OA model.

## Materials and methods

### Screening of FDA-approved drugs with TOPFlash reporter assay

Human chondrosarcoma (HCS-2/8) cells, which were kindly provided by Dr. Masaharu Takigawa at Okayama University [31], were cultured in Dulbecco’s Modified Eagle’s Medium (DMEM, Thermo Fisher Scientific) supplemented with 10% fetal bovine serum (FBS, Thermo Fisher Scientific). The conditioned medium enriched with mouse Wnt3A (Wnt3A-CM) was harvested from L Wnt-3A cells (CRL-2647, ATCC), which stably express mouse *Wnt3a*, according to the manufacturer’s protocol. Briefly, L Wnt-3A cells were cultured in 10 ml DMEM supplemented with 10% FBS in a 10-cm dish, and were grown to confluency. The cells were cultured two additional days, and the medium was harvested as the first batch. Ten ml of fresh culture medium were added to the cells, and harvested 2 days later as the second batch. The first and second batches were mixed in a ratio of 1:1 to make Wnt3A-CM. We screened 1,186 FDA-approved chemical compounds (Prestwick Chemical) for reduction of Wnt/β-catenin signaling activated by 25% Wnt3A-CM. We used TOPFlash firefly luciferase reporter plasmid, which has eight TCF/LEF-binding sites in pTA-Luc vector (M50 Super 8 × TOPFlash plasmid, Addgene) to measure β-catenin-mediated transcriptional activation in HCS-2/8 cells treated with Wnt3A-CM [29]. HCS-2/8 cells were seeded in a 100-mm dish (Thermo Fisher Scientific) and cultured for 2–3 days up to 60–70% confluency. Then, cells were transfected with TOPFlash firefly luciferase reporter plasmid (5.5 μg/dish) and phRL-TK encoding Renilla luciferase (0.5 μg/dish, Promega) using FuGENE 6 (Thermo Fisher Scientific) according to the manufacturer’s protocols. At 24 hours after transfection, the cells were seeded at 1 × 10^4^ cells/well in a 96-well culture plate (Falcon) in the presence of 10 μM each drug and 25% Wnt3A-CM, and were cultured for 24 additional hours. Luciferase activity was measured using the Dual Luciferase Reporter Assay System (Promega) and PowerScan MX (DS Parma Biomedical). Firefly luciferase activity was normalized by Renilla luciferase activity, and relative luciferase units (RLU) were calculated.

### Isolation of human osteoarthritic chondrocyte (OAC) cells

Human studies were approved by the Ethical Review Committee of the Nagoya University Graduate School of Medicine. Human OAC cells were isolated from the dissected cartilage in patients with knee OA at the time of joint replacement surgery after appropriate written informed consent was obtained. Articular cartilage from the femoral condyles, tibial plateaus, and articular surface of the patella was cut into small pieces with a scalpel. Cartilage samples were minced and digested at 37°C with trypsin–EDTA solution for 30 min, incubated with 3 mg/ml type II collagenase (Worthington Biochemical Co.) in Dulbecco’s Modified Eagle’s Medium (DMEM, Thermo Fisher Scientific) for 18 hours, filtered through a nylon mesh, and washed extensively. Next, the isolated chondrocytes were seeded in 75 cm^2^ culture flasks and incubated in DMEM containing 10% fetal bovine serum, penicillin (100 U/ml), and streptomycin (100 mg/ml) at 37°C in an atmosphere of 5% CO_2_. At confluence, the cells were detached and seeded in a 12-well plate at a density of 2 × 10^5^/well [32]. OAC cells were cultured in either a 12-well plate or a Cell Culture Chamber Slide (Thermo Fisher Scientific), and treated with 25% Wnt3A-CM, 10 mM LiCl and/or 10 μM fluoxetine for 48 hours.

We confirmed that Wnt3A-CM treatment upregulated *AXIN2*, and downregulated *SOX9* and *MMP13* in OAC cells (S1 Fig). The opposite effects of Wnt3A-CM on MMP13 in mouse ATDC5 cells and human OAC cells have been previously reported [13], but the underlying mechanisms remain to be elucidated.

### Generation of a rat OA model

All animal studies were approved by the Animal Care and Use Committee of the Nagoya University. Sprague Dawley (SD) rats (8 weeks old) were anesthetized with an intraperitoneal injection of pentobarbital sodium (0.1 ml of 10 mg/ml). Under sterile conditions, the right knee of the rat was induced to OA by resection of the menisco-tibial ligament to destabilize medial meniscus (DMM surgery) [33]. Skin and joint capsule on the left knee were incised and sutured (Sham operation). We dissolved indicated concentrations of fluoxetine in PBS for intraarticular injections. Fifty μl of 0, 50, 100, or 200 μM fluoxetine in PBS was intraarticularly injected into both knees once a week. After 8 postoperative weeks, rats were sacrificed and tissues around the knees were fixed overnight in 4% paraformaldehyde at 4°C, dehydrated, and embedded in paraffin. As previously reported [33][34], we also observed that the section at the medial compartment comprised of the medial femoral condyle and the medial tibial plateau (i.e. the weight-baring area) showed the severest OA damage with the highest Mankin score in DMM rats. We thus made ~10 sections around the medial compartment, and analyzed a section with the narrowest interarticular space. To ensure that the entire chondral surface is similar to what we observed at the medial compartment, we also analyzed a section with the narrowest interarticular space at the lateral compartment.

### Evaluation of OA progressions in a rat OA model with modified Mankin score

Sections at the medial compartment (the medial femoral condyle and the medial tibial plateau) or the lateral compartment (the lateral femoral condyle and the lateral tibial plateau) in a rat model were stained with Safranin O and Fast-green. OA progressions were graded according to the modified Mankin histologic score on both tibial and femoral sides of articular cartilage [35, 36]. The modified Mankin score is a sum of the following seven parameters: articular cartilage structure, grades 0–11; tidemark duplication, grades 0–3; Safranin O staining, grades 0–8; fibro-cartilage, grades 0–2; chondrocyte clones in uncalcified cartilage, grades 0–2; hypertrophic chondrocytes in calcified cartilage, grades 0–2; and subchondral bone, grades 0–2. The grades of OA were estimated by a single blinded observer and averaged in each group of mice.

### Immunostaining of articular cartilage in a rat OA model

We confirmed that, unlike a mouse rheumatoid arthritis (RA) model [37], immunostaining of TNF and IL6 was not increased in DMM surgery (Panels A and B in S2 Fig). Similarly, 10 mM LiCl had no substantial effect on expressions of TNF and IL6 in OAC cells (Panel C in S2 Fig). Sections with the narrowest interarticular space at the medial and lateral compartments were immunostained for β-catenin. First, the paraffin-embedded sections were deparaffinized and rehydrated. Then, the sections were unmasked by 10 mM sodium citrate buffer (pH 6.0) at 90°C for 15 min and were incubated with 3% H_2_O_2_ for 15 min to inactivate endogenous peroxidases. Sections were blocked with 5% goat serum in TBS-T for 1 hour at room temperature followed by overnight incubation with antibodies against β-catenin (dilution 1:100), TNF, or IL6 (dilution 1: 100 in 5% goat serum with TBS-T, #9587, CST) at 4°C. After washing with TBS-T, sections were incubated with Alexa 488-conjugated donkey anti-rabbit IgG (dilution 1: 500, Thermo Fisher Scientific) for 1 hour at room temperature. Finally, the specimens were mounted in VectaShield containing 2 μg/ml diamidino-2-phenylindole (DAPI, Vector Laboratories) and visualized using the IX71 (Olympus) microscope.

### Quantification of β-catenin signals in articular chondrocytes

We evaluated cytoplasmic and nuclear accumulation of β-catenin in articular chondrocytes using the MetaMorph software (Molecular Device). We analyzed the entire articular chondrocytes except for the areas where articular cartilage was severely damaged and no or only a few chondrocytes were observed. We quantified ~200–300 μm^2^ for each section. Signals less than 4 μm in diameter were discarded as non-specific signals, and signals more than 4 μm in diameter were taken as positive signals. DAPI-staining was used to localize the nucleus and to count the number of cells. When the intensity of β-catenin in the nucleus was similar to or more than that in the cytoplasm, the cell was counted as a nuclear β-catenin-positive cell. All quantification was performed by a blinded observer.

### Statistical analysis

The continuous variables are expressed as mean ± standard deviation (SD). The comparison between two conditions was carried out using Student’s *t*-test. The comparison among multiple groups was carried out using one-way analysis of variance (ANOVA) and followed by Tukey-Kramer test. All statistical analyses were performed with SPSS Statistics 23 (IBM) and SAS version 9.4 (SAS Institute, Inc., Cary, NC, USA).

### Other materials and methods

Details of other materials and methods are available in Supplementary Information, which includes the following methods: Alcian blue staining, total RNA extraction and real-time RT-PCR analysis, Western blotting, co-immunoprecipitation assay for Axin1, immunofluorescence staining of OAC cells, and MTS assay.

## Results

### Fluoxetine reduces the Wnt/β-catenin signaling activity in HCS-2/8 cells

**Fig 1:**
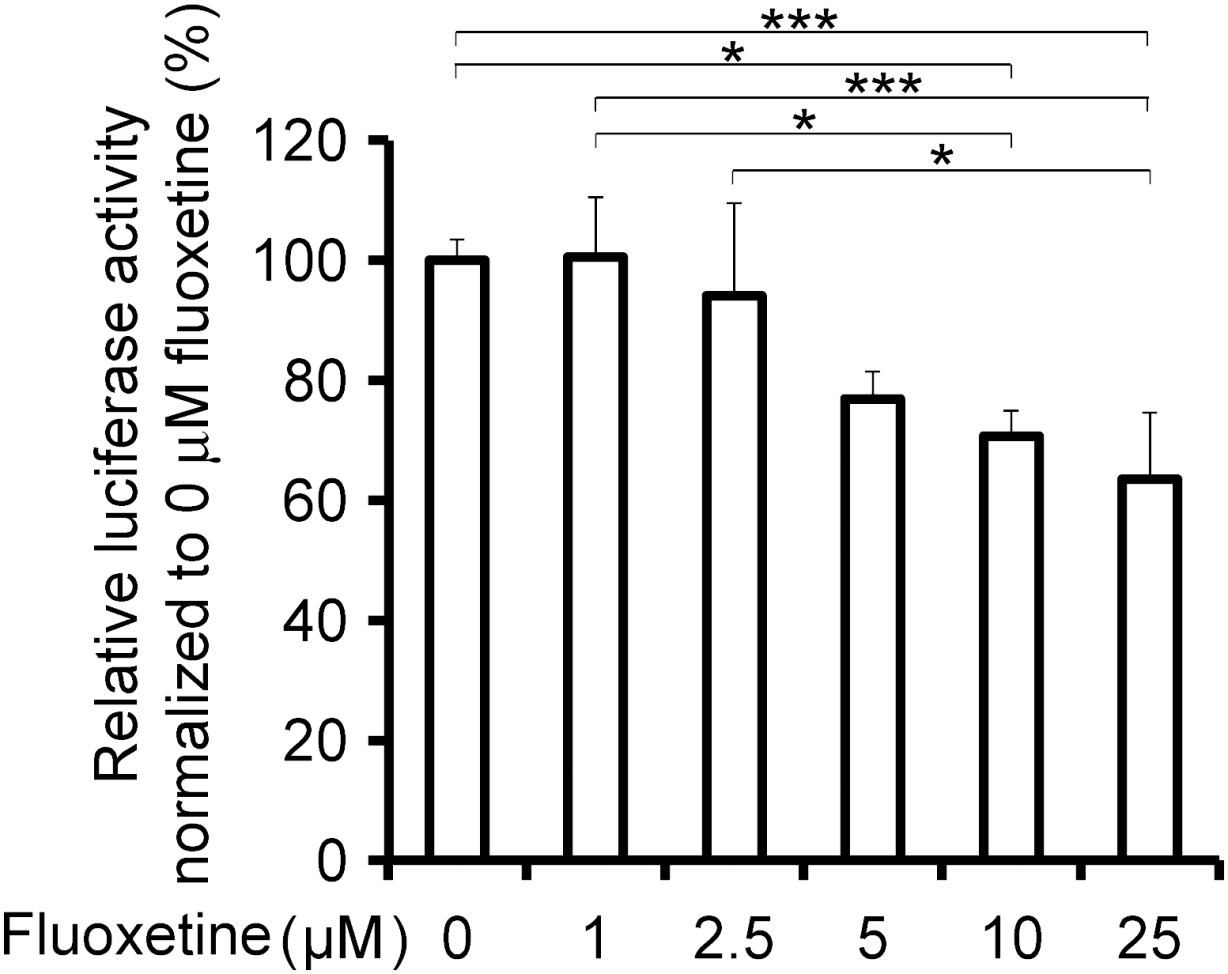
Fluoxetine decreases TOPFlash firefly luciferase reporter activity. HCS-2/8 human chondrosarcoma cells transfected with TOPFlash are treated with 25% conditioned medium containing Wnt3A (Wnt3A-CM) and increasing concentrations of fluoxetine for 24 hours. Firefly luciferase activity is normalized to the TK promoter-driven Renilla luciferase activity and to 0 μM fluoxetine. The mean of six wells in a single experiment is calculated first. Then, the means of six wells in three independent experiments are used to calculate the mean and SD (*n* = 3). *P*-value by one-way ANOVA is 0.00076. *p < 0.05 and ***p < 0.005 by posthoc Tukey-Kramer test.

To identify a clinically applicable drug for OA, chondrogenic HCS-2/8 human chondrosarcoma cells [31] were transfected with the TOPFlash reporter plasmid, which quantifies the Wnt/β-catenin activity. We searched for a drug that suppresses the Wnt/β-catenin signaling activated by 25% Wnt3A-CM among FDA-approved drugs. We found that 10 μM fluoxetine, an antidepressant in the class of selective serotonin reuptake inhibitors (SSRI), showed 33 ± 2.2% (mean and SD, *n* = 5) reduction of relative TOPFlash reporter activity. In addition, fluoxetine suppressed the TOPFlash reporter activity in a dose-dependent manner (Fig 1). We also examined two other SSRIs. In contrast to fluoxetine, the relative TOPFlash reporter activities were not suppressed by 10 μM paroxetine and 10 μM fluvoxamine: 5.4 ± 18.7% reduction and 23 ± 38.0% increase (mean and SD, *n* = 5), respectively. Thus, the inhibitory effect on TOPFlash by fluoxetine is unlikely due to its inhibitory effect on serotonin reuptake. As fluoxetine is the first anti-depressant in the class of serotonin-reuptake inhibitor and has been long used in clinical practice [38], we pursued the effect of fluoxetine in the current study.

### Fluoxetine attenuates Wnt3A- and LiCl-induced degradation of proteoglycans in differentiated ATDC5 cells

**Fig 2:**
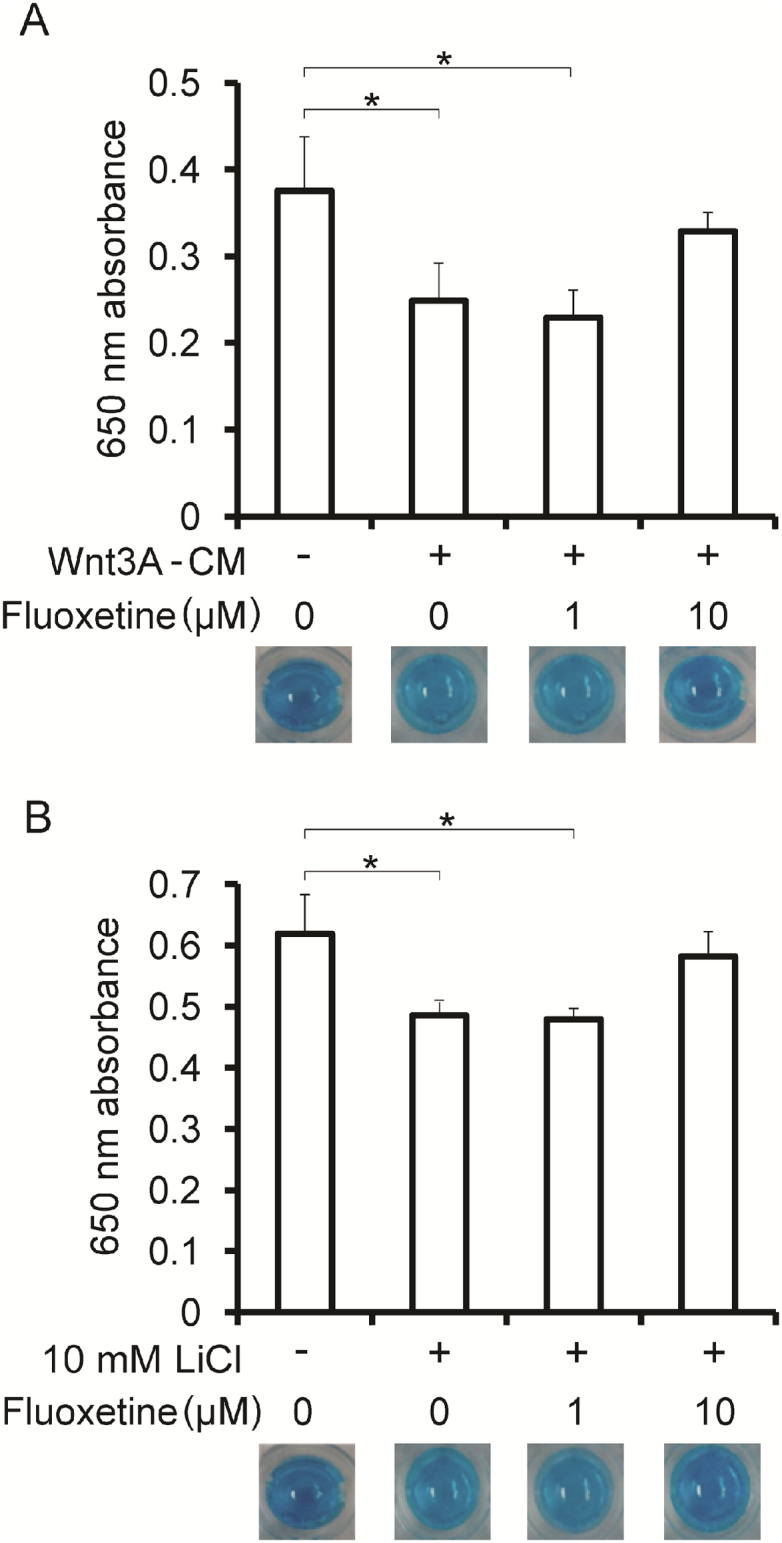
Fluoxetine attenuates Wnt3A- and LiCl-induced loss of proteoglycans in chondrogenically differentiated ATDC5 cells. Alcian blue staining of ATDC5 cells that are differentiated to chondrocytes with ITS for 2 weeks. The cells are subsequently treated with 20% Wnt3A-CM **(A)** and 10 mM LiCl **(B)** in the presence of indicated concentrations of fluoxetine for 48 hours. Proteoglycans are quantified by measuring the optical density at 650 nm of the cell lysates. The assay is performed in three wells in a single experiment, and representative Alcian blue stainings are indicated. The mean and SD (*n* = 3 wells) are indicated. *P*-values by one-way ANOVA of each condition are 0.011 **(A)** and 0.010 **(B)**, respectively. *p < 0.05 by posthoc Tukey-Kramer test.

In articular cartilage, chondrocytes are embedded in an abundant extracellular matrix composed of proteoglycans and collagens. Mouse ATDC5 cells recapitulate chondrogenic differentiation, and produce more proteoglycans than HCS-2/8 cells [39]. We thus investigated the effect of fluoxetine on production of proteoglycans in differentiating ATDC5 cells. LiCl is an inhibitor of GSK3 and activates Wnt/β-catenin signaling [40] in a plethora of cell lines at 10 mM concentration. As predicted, Alcian blue staining showed that LiCl and 20% Wnt3A-CM reduced the amounts of proteoglycans in differentiated ATDC5 cells to statistically significant levels. We found that 10 μM fluoxetine extinguished the statistical significance, and resumed the amounts of proteoglycans but not to the levels without Wnt3A-CM or LiCl (Fig 2).

### Fluoxetine upregulates *Sox9* mRNA, and downregulates *Axin2* and *Mmp13* mRNAs in differentiated ATDC5 cells

**Fig 3:**
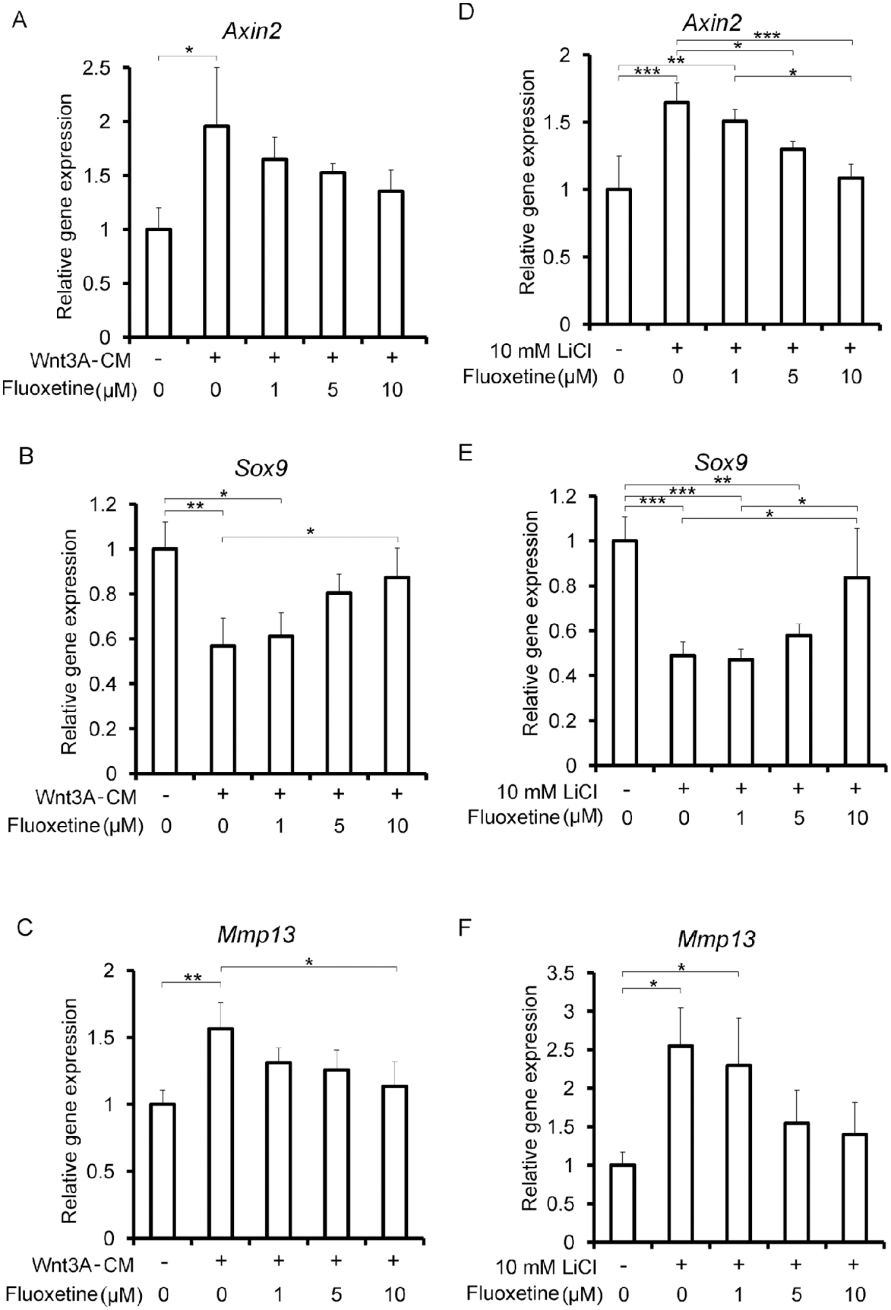
Fluoxetine upregulate chondrogenic *Sox9* and tends to downregulate Wnt-responsive *Axin2* and ECM-degrading *Mmp13* in chondrogenically differentiated ATDC5 cells. ATDC5 cells are treated with ITS to induce chondrogenic differentiation. Differentiated ATDC5 cells are treated with 20% conditioned medium containing Wnt3A (Wnt3A-CM) **(A, B, and C)** or 10 mM LiCl **(D, E, and F)** for 48 hours. Expression levels of each mRNA are normalized to that without treatment. The mean value of three wells in a single experiment is calculated first. Then, the means of three wells in three independent experiments are used to calculate the mean and SD (*n* = 3). *P*-values by one-way ANOVA are 0.028 **(A)**, 0.0038 **(B)**, 0.0089 **(C)**, 0.00070 **(D)**, 0.00090 **(E)**, and 0.0099 **(F)**, respectively. *p < 0.05, **p < 0.01, and ***p < 0.005 by posthoc Tukey-Kramer test.

We next confirmed in chondrogenically differentiated ATDC5 cells that 20% Wnt3A-CM (Fig 3A, 3B and 3C), recombinant mouse Wnt3A protein (Panel C in S3 Fig), and LiCl (Fig 3D, 3E and 3F) (i) upregulated *Axin2* mRNA, a specific marker for Wnt/β-catenin signaling, (ii) decreased *Sox9* mRNA, a master gene for chondrocyte differentiation, and (iii) upregulated *Mmp13* mRNA, coding for a proteinase for collagen type II. Similarly, LiCl upregulated *Matn1* mRNA, encoding one of extracellular cellular matrix proteins upregulated by Sox9 [41], in chondrogenically differentiated ATDC5 cells (Panel A in S4 Fig).

We found that fluoxetine partly rescued down-regulation of *Sox9* induced by 20% Wnt3A-CM (Fig 3B) and LiCl (Fig 3E). Similarly, fluoxetine partly ameliorated down-regulation of *Matn1* induced by LiCl in a dose-dependent manner (Panel A in S4 Fig). In addition, Wnt-induced upregulations of *Axin2* (Fig 3A and 3D) and *Mmp13* (Fig 3C and 3F) were partly rescued by fluoxetine with (Fig 3C and 3D) or without (Fig 3A and 3F) statistical significance. Although no statistical significance was observed in Fig 3A and 3F, statistically significant increases of *Axin2* and *Mmp13* were attenuated by 10 μM fluoxetine to the levels without statistical significance.

### Fluoxetine stabilizes the β-catenin degradation complex and induces β-catenin degradation in differentiated ATDC5 cells

**Fig 4:**
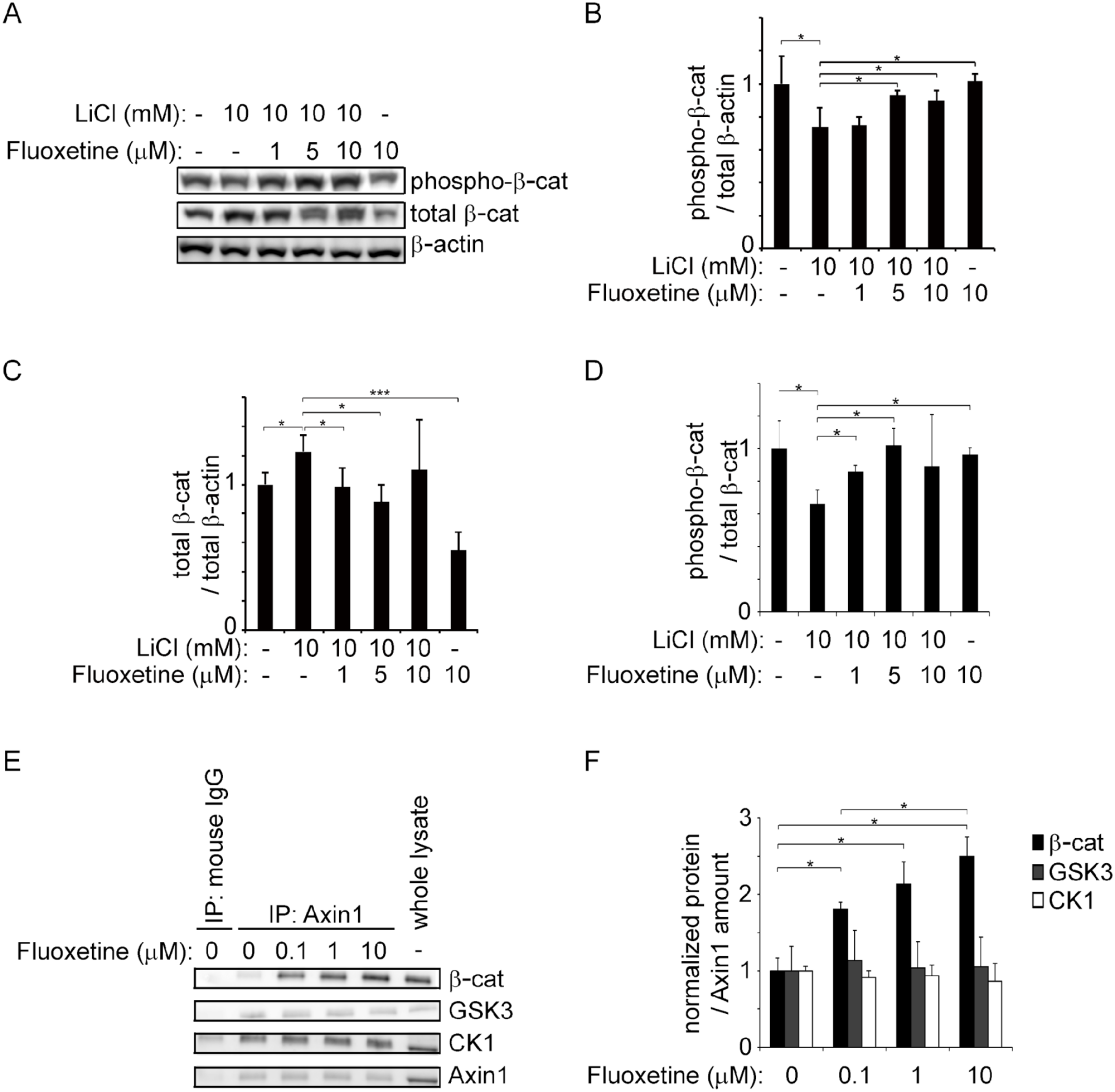
Fluoxetine increases the ratio of phosphorylated β-catenin-to-β-actin, and enhances binding of Axin1 to β-catenin. **(A-D)** Immunoblotting of total and phosphorylated β-catenin (β-cat) in differentiated ATDC5 cells treated with LiCl and fluoxetine for 48 hours. An antibody recognizes either total β-catenin or β-catenin phosphorylated at serine 33, serine 37, and threonine 41. Phosphorylation of β-cat at these residues triggers ubiquitination and degradation of β-cat [7]. The ratios of phosphorylated-to-total β-actin, total β-cat-to-total β-actin, and phosphorylated β-cat-to-total β-cat are normalized to those without LiCl or fluoxetine. The mean and SD in three independent experiments are indicated. *P*-values by one-way ANOVA are 0.00063 for phosphorylated β-cat-to-total β-actin **(B)**, 0.00091 for total β-cat-to-total β-actin **(C)** and 0.00083 for phosphorylated-to-total β-cat **(D)**. Statistical differences between 10 mM LiCl without fluoxetine (the second bar) and the others are indicated. *p < 0.05 and ***p < 0.005 by posthoc Tukey-Kramer test. **(E and F)** Immunoprecipitation of β-catenin, GSK3, and CK1 with anti-Axin1 antibody in whole lysates of L cells. The whole lysates were added with indicated concentrations of fluoxetine. Band intensities are normalized to that of Axin1, and also to that without fluoxetine. As a control, whole lysate is immunoprecipitated with normal mouse IgG. The mean and SD in three independent experiments are indicated. *P*-values by one-way ANOVA are 0.018 for β-catenin, 0.14 for GSK3, and 0.38 for CK1. *p < 0.05, **p < 0.01, and ***p < 0.005 by posthoc Tukey-Kramer test.

We next examined the effect of fluoxetine on β-catenin (Fig 4A). In differentiated ATDC5 cells, LiCl reduced the level of phospho-β-catenin (Ser33/Ser37/Thr41) compared to total β-actin (Fig 4B). As phosphorylation of β-cat at Ser33/Ser37/Thr41 physiologically causes degradation of β-cat [7], LiCl attenuated degradation of total β -cat (Fig 4C) and reduced the ratio of phospho- β-catenin to total β-catenin (Fig 4D). We found that fluoxetine partly mitigated LiCl-induced suppression of phospho-β-catenin in a dose dependent manner (Fig 4A, 4B and 4D). We also found that 1 and 5 μM fluoxetine partly mitigated LiCl-induced induction of total β-catenin, but we observed no effect at 10 μM fluoxetine (Fig 4C). As the effect of LiCl is inhibition of GSK3, which phosphorylates Ser33/Ser37/Thr41 of β-catenin, we hypothesized that the direct target of fluoxetine is the β-catenin degradation complex comprised of GSK3, Axin1, and CK1. CK1 is another kinase for β-catenin and phosphorylates Ser45. Axin1 binds to β-catenin and its kinases, GSK3 and CK1, in a degradation complex, and functions as a scaffold for the β-catenin degradation complex. To examine a direct *in vitro* effect of fluoxetine on the degradation complex, we immunoprecipitated Axin1 *in vitro* with an antibody against Axin1 in the presence of 0.1, 1.0, and 10 μM fluoxetine, and detected coimmunoprecipitated β-catenin, GSK3, and CK1 (Fig 4E and 4F). For harvesting enough amounts of those proteins [42], we used cell extracts of L cells. Fluoxetine had no effect on coimmunoprecipitation of GSK3 or CK1. In contrast, fluoxetine increased the amount of coimmunoprecipitated β-catenin in a dose-dependent manner. Thus, fluoxetine enhances binding of β-catenin to the degradation complex, and subsequently facilitates β-catenin degradation.

Although GSK3 was unlikely to be a direct target of fluoxetine, GSK3 functions not only downstream of Wnt3A but also downstream of growth hormone via Akt1/2 in rat growth plate [43]. We thus examined whether Akt1/2 was activated in differentiated ATDC5 cells, and found that Akt1/2 inhibitor had no effects on *Axin2*, *Sox9* and *Mmp13* expressions (Panel B in S4 Fig).

### Fluoxetine suppresses accumulation of β-catenin in human osteoarthritic chondrocyte (OAC) cells

**Fig 5:**
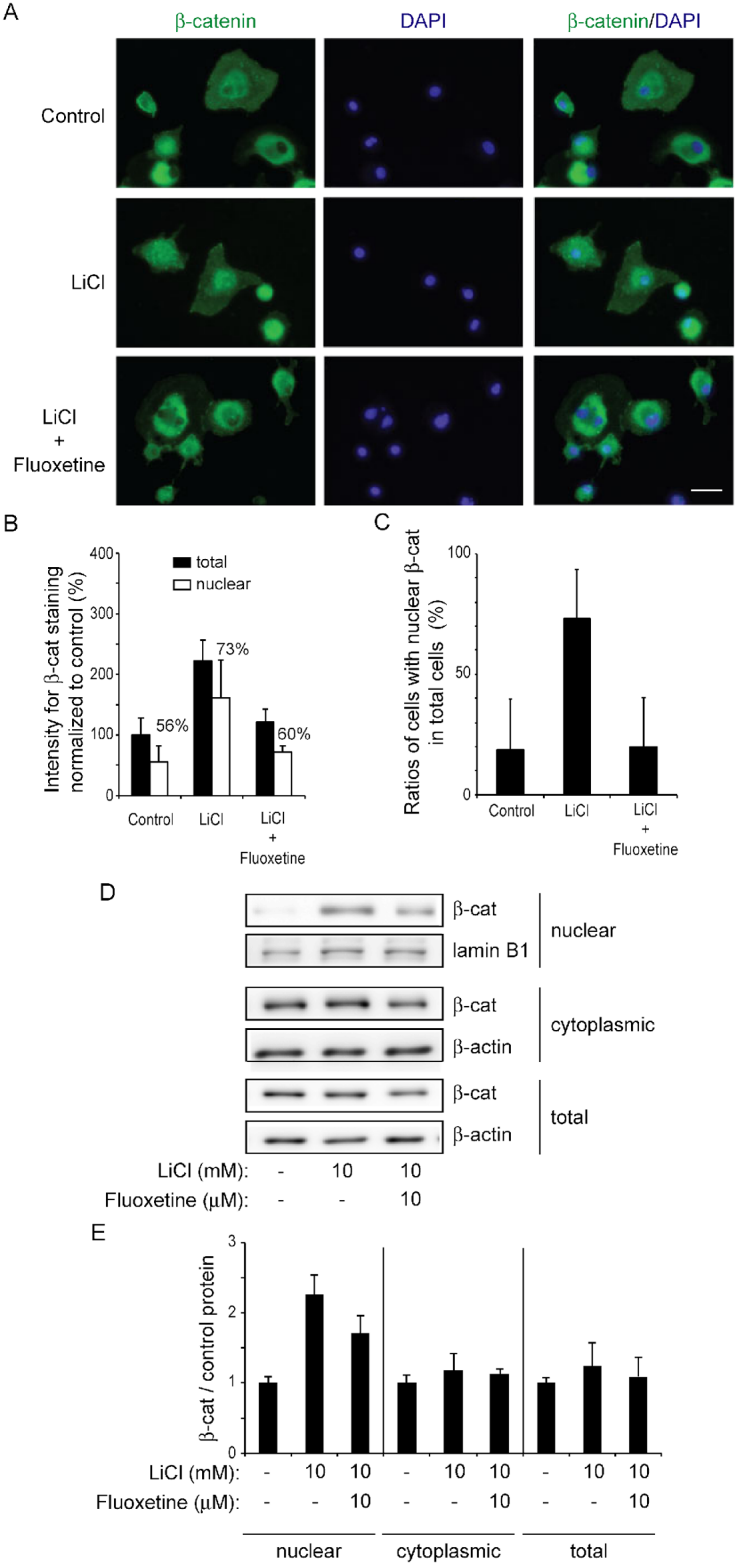
Fluoxetine tends to suppress LiCl-mediated β-catenin accumulation in human osteoarthritic chondrocyte (OAC) cells. **(A)** Representative images for immunofluorescence staining with anti-β-catenin antibody in OAC cells. Untreated cells (Control), cells treated with LiCl (LiCl), and cells treated with LiCl and 10 μM fluoxetine (LiCl + fluoxetine) for 48 hours are stained with anti-β-catenin antibody (green) and DAPI (blue). Scale bar = 40 μm. **(B)** Intensities of β-catenin (β-cat) staining in the total cellular area and the DAPI-positive nuclear area are blindly analyzed with MetaMorph and normalized to the number of cells and also to that of control without LiCl and fluoxetine (*n* = 2 patients). Ratios of nuclear β-catenin signal (open bar) divided by total β-catenin signal (closed bar) in each group are indicated above each bar. The mean and SD (*n* = 2 patients) are indicated. **(C)** When the intensity of β-catenin in the nucleus is similar to or more than that in the cytoplasm, the cell is counted as a nuclear β-catenin-positive cell using MetaMorph. The mean and SD (*n* = 2 patients) are indicated. **(D, E)** Immunoblotting with anti-β-catenin antibody of nuclear and cytoplasmic fractions of OAC cells. Cells treated with 10 mM LiCl with or without 10 μM fluoxetine for 48 hours are lysed for collecting each fraction. The immunoblots are performed in 4 wells each in two different experiments (*n* = 8). Representative immunoblots are shown. Densities of β-catenin/lamin B1 (a nuclear fraction) and β-catenin/β-actin (a cytoplasmic or total fraction) are normalized to a fraction without LiCl or fluoxetine, and are plotted in **E**. The mean and SD (*n* = 2 patients) are indicated.

To examine whether fluoxetine has an effect on Wnt/β-catenin signaling in human OA, we analyzed the effect of fluoxetine on primary OAC cells isolated from knee joints of two OA patients. Immunofluorescence staining of β-catenin showed that fluoxetine tended to suppress the intensity of β-catenin staining per OAC cell in both the nucleus and the cell body (Fig 5A, 5B and 5C). Immunoblotting of the nuclear fraction additionally showed that fluoxetine tended to decrease β-catenin localization in the nucleus, which was induced by LiCl treatment (Fig 5D and 5E). Finally, quantitative RT-PCR showed that fluoxetine tended to reduce expression of *AXIN2* in OAC cells, as we observed in ATDC5 cells (S2 Fig).

### Fluoxetine reduces cartilage degradation in a rat OA model

**Fig 6:**
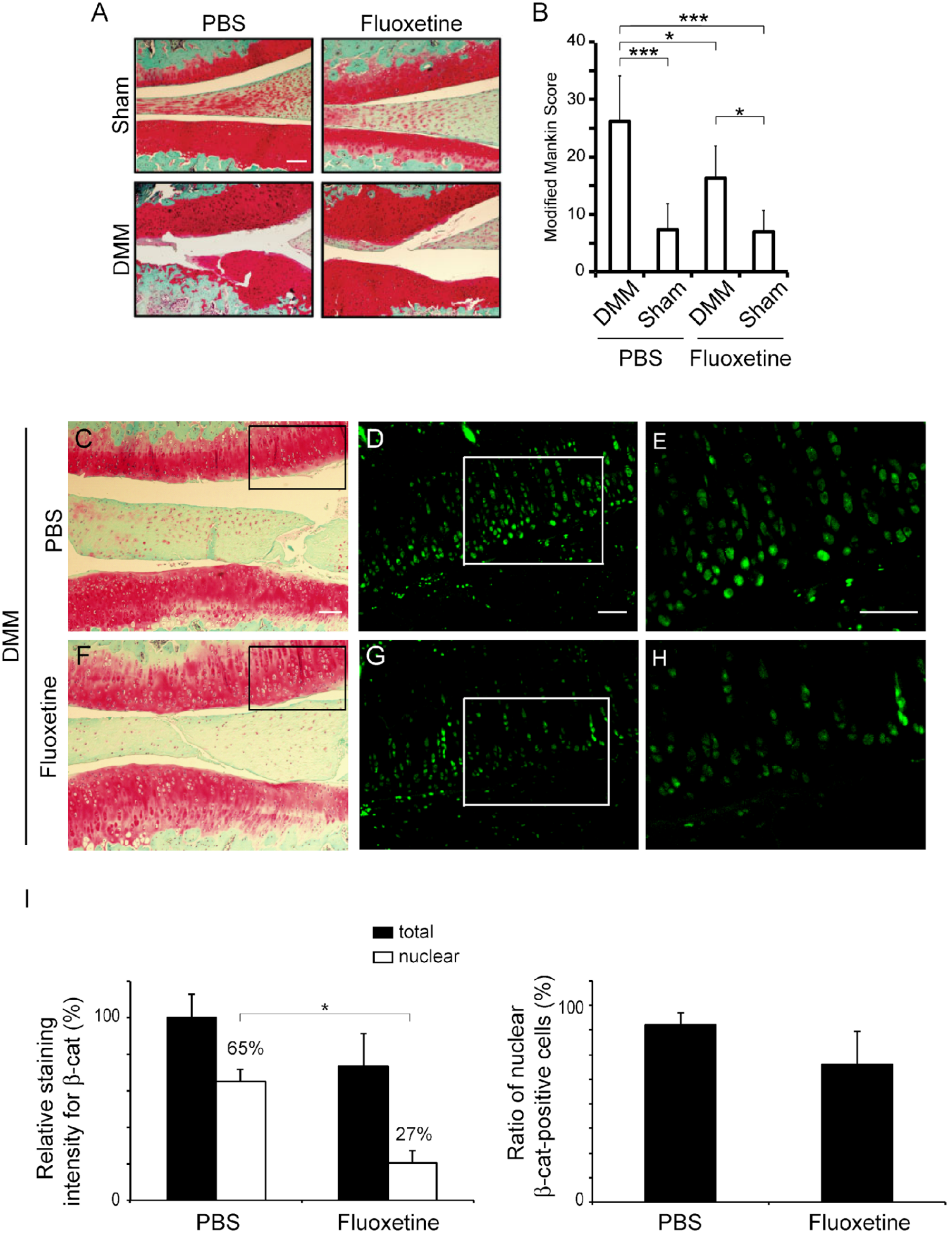
DMM surgery induces mild OA phenotype in SD rat and 50 μM fluoxetine prevents OA progression. Surgical destabilization of medial meniscus (DMM surgery) is performed on the right knees of 12 SD rats. On the left knees of these rats, skin and joint capsule are incised and sutured (Sham). PBS (50 μl) (*n* = 6 rats) or 50 μM fluoxetine dissolved in 50 μl PBS (*n* = 6 rats) is intraarticularly injected into both knees once a week for 8 weeks after operation. We made ~10 sections around the medial compartment for each rat, and analyzed a single section with the narrowest interarticular space. **(A)** Representative staining of knee joints at the medial compartment comprised of the medial femoral condyle and the medial tibial plateau with Safranin O and Fast-green. **(B)** Fluoxetine suppresses OA progressions evaluated by modified Mankin score at 8 weeks after the surgery at the medial compartment. The mean and SD (*n* = 6 rats) are indicated. *P*-value by one-way ANOVA is < 0.0001. *p < 0.05 and ***p < 0.005 by posthoc Tukey-Kramer test. Modified Mankin scores evaluated with sections at the lateral compartments are shown in Panel A in S6 Fig **(C, F)** Representative staining of knee joints at the medial compartments with Safranin O and Fast-green. Sections with the narrowest interarticular space at the medial and lateral compartment are immunostained for β-catenin. Although Safranin O staining in **F** is fainter than that in **C**, fluoxetine is predicted to have no effect on Safranin O staining, because fluoxetine- and PBS-treated cartilages are stained with Safranin O to a similar extent in **A** and **Panel B in** S6 Fig **(D, G)** Articular chondrocytes are immunostained with anti-β-catenin antibody. Serial sections of boxed areas in C and F are shown. **(E, H)** Magnified images of boxed areas in D and G. Larger areas of stained sections in A, C and F are shown in Panel B in S6 Fig **(I, J)** Blinded morphometry of β-catenin signals of the chondrocytes at the medial and lateral compartments with MetaMorph. **(I)** Signal intensities for β-catenin in the total cellular area and the DAPI-positive nuclear area are normalized to the number of cells and also to that in total cellular area in PBS-treated cartilage. *P*-values by Student *t*-test are 0.078 for total signals and 0.038 for nuclear signals (*n* = 4 knees in each group). *p < 0.05. **(J)** The number of β-catenin-positive cells is divided by the number of DAPI signals to calculate the ratio of nuclear β-catenin-positive cells. *P* = 0.060 by Student *t*-test (*n* = 4 knees in each group). **(A, C)** Scale bar = 200 μm. **(D, E)** Scale bar = 100 μm.

We next analyzed the effects of fluoxetine on progression of OA in a rat model. We performed surgical destabilization of the medial meniscus (DMM surgery) in SD rats, and histologically evaluated progression of OA. To examine the effects of fluoxetine on articular chondrocytes *in vivo*, rats were intraarticularly injected with 50 μl of 50 μM (Fig 6), 100 μM (S5 Fig), and 200 μM (S5 Fig) fluoxetine once a week for 8 weeks after the surgery. As a control, we intraarticularly injected PBS once a week for 8 weeks. The section crossing the narrowest interarticular portion at the medial compartment comprised of the medial femoral condyle and the medial tibial plateau showed the severest OA damage with higher Mankin score in DMM rat than the other sections, as previously reported [33, 34]. We thus stained the narrowest interarticular section with Safranin O and Fast-green for evaluation of OA (Fig 6A–6C, 6F and S5 Fig) Only 50 μM fluoxetine lowered the modified Mankin score, whereas 100 μM fluoxetine minimally lowered the score. In contrast, 200 μM fluoxetine damaged the joints of DMM-operated knees, as well as sham-operated knees (S5 Fig). The difference in the modified Mankin scores in similarly treated PBS-injected DMM-operated knees between Fig 6 and S5 Fig was likely due to inter-batch variability. As the standard deviations of the modified Mankin scores of intra-batch knees were low in both Fig 6 and S5 Fig, we expected that 50 μM fluoxetine has the most effects compared to the other concentrations.

The recommended oral doses of fluoxetine for depression in human are 0.33–1.33 mg/kg/day (20–80 mg/60 kg human/day). In a previous report, intraperitoneal injection of 25 mg/kg/day (0.625 mg/25 g mouse/day) fluoxetine, which was 19–75 times higher than human doses, exhibited potent anti-inflammatory activity in a mouse model of rheumatoid arthritis [44]. Assuming that 20–80 mg of fluoxetine is distributed throughout the human body fluid including the synovial fluid, the concentration is predicted to be 20–80 mg/MW 309.3/(60 kg x 0.6) = 1.8–7.2 μM. Thus, 50 μM fluoxetine in the synovial fluid was 7–28 times higher than the calculated concentrations in the human body fluid immediately after injection, but fluoxetine was predicted to be diffused into extra-articular space and not to stay in the synovial fluid for a long time. At 8 weeks after operation, articular cartilage of the sham-operated knee was evenly stained with Safranin O in both the PBS-injected and fluoxetine-injected groups (Fig 6A and 6B). In contrast, articular cartilage of the DMM-operated knee showed moderate fibrillation, cleft, cartilage loss, and moderate loss of Safranin O staining. On the DMM-operated side, the modified Mankin score at the medial compartment (i.e. the weight-baring area) was 26.3 ± 8.0 (mean ± SD, *n* = 6 rats) in the PBS group and 16.3 ± 5.7 in the fluoxetine group (Fig 6B). In a section crossing the narrowest interarticular portion at the lateral compartment, the modified Mankin score was 5.75 ± 3.0 (n = 4 rats) in the PBS group and 2.0 ± 1.6 in the fluoxetine group (Panel A in S6 Fig) Immunofluorescence staining for β-catenin (Fig 6C–6H) and its quantitative analysis (Fig 6I) showed that fluoxetine reduced the intensity of β-catenin significantly in the nucleus and marginally in the cell body at the medial and lateral compartments. In addition, the numbers of cells with positive nuclear β-catenin signals were slightly reduced by fluoxetine treatment. These results suggest that intraarticular administration of fluoxetine ameliorates OA progression in a rat OA model.

## Discussion

In contrast to rheumatoid arthritis (RA), no disease-modifying drugs are currently available for OA [45]. Analgesics and anti-inflammatory agents, such as acetaminophen and cyclooxygenase (COX) inhibitors, are prescribed as symptomatic treatment for OA. Another form of local administration to relieve pain in OA patients is intraarticular injection of hyaluronic acid and corticosteroids. In severe OA cases, joint replacement surgery is required.

We previously reported that verapamil, a calcium channel blocker, protected against cartilage degradation by inducing expression of FRZB in a rat OA model [29]. FRZB is a secreted Wnt antagonist. We identified verapamil by measuring the promoter activity of *FRZB* in cultured cells. Verapamil thus suppresses Wnt/β-catenin signaling extracellularly, and should have no direct intracellular effect. In addition to activation of β-catenin, Wnt ligands also activate non-canonical PCP and Ca^2+^ pathways [25], the roles of which in OA, however, have not been dissected. To search for another preapproved drug that specifically suppresses Wnt/β-catenin signaling, we used the TOPFlash reporter assay and identified fluoxetine. Fluoxetine had no effect on the promoter activity of *FRZB* [29]. Instead, in contrast to verapamil, fluoxetine was able to attenuate LiCl-induced activation of Wnt/β-catenin signaling in chondrogenically differentiated ATDC5 cells. LiCl is a GSK3α/β inhibitor and increases the amount of β-catenin by blocking phosphorylation and subsequent degradation of β-catenin. Fluoxetine was thus predicted to work downstream of GSK3. Indeed, fluoxetine increased a binding affinity of Axin1 with β-catenin, but not with GSK3 or CK1. As Axin1 is a scaffold protein for forming the degradation complex comprised of β-catenin, GSK3, and CK1, fluoxetine is likely to stabilize binding of β-catenin to the degradation complex, and to enhance phosphorylation and subsequent degradation of β-catenin in chondrocytes. Lack of the suppressive effect of Wnt/β-catenin signaling in other SSRIs, paroxetine and fluvoxamine, also supports the notion that the suppressive effect of fluoxetine on Wnt/β-catenin signaling is not due to its SSRI effect.

Wnt/β-catenin signaling is important for cell proliferation, differentiation, and survival in adult tissues. We showed here that fluoxetine reduced loss of ECM (Fig 2) and expression of *Mmp13* (Fig 3) in ATDC5 cells treated with Wnt3A-CM and LiCl. However, fluoxetine did not perturb a basal level of β-catenin phosphorylation in ATDC5 cells (Fig 4A). In addition, intraarticular administration of fluoxetine inhibited OA progression in the DMM-operated knee, but had no effect on integrity of the cartilage in the sham-operated knee (Fig 6). Suppression of Wnt/β-catenin signaling by fluoxetine is thus unlikely to have an unexpected adverse effect on normal cartilage.

Most antidepressants are able to relieve pain [46]. Indeed, duloxetine, an antidepressant in the class of selective serotonin-norepinephrine reuptake inhibitor (SNRI), alleviates pain in OA patients [47]. To the best of our knowledge, no report has addressed the effect of fluoxetine on OA. This was likely because alleviation of OA pain by fluoxetine might have been attributed to its pain-relieving effect. Another possibility is that only intraarticular, but not oral, administration of fluoxetine exerts its effect. Alternatively, psychiatrists who prescribed fluoxetine might have had no chance to deeply look into features related to OA. Retrospective studies of OA progression in patients with depression taking fluoxetine will be able to disclose whether oral administration of fluoxetine has beneficial effects on OA. Antidepressants have fewer side effects in the long term than traditionally prescribed analgesics including opioids and non-steroidal anti-inflammatory drugs (NSAIDs). Fluoxetine has been widely used since its approval by FDA in 1986. Fluoxetine is expected to relieve OA-associated pain in addition to its suppressive effect on abnormally activated Wnt/β-catenin signaling in OA patients.

## Supporting information

S1 Fig Responses on *AXIN2*, *SOX9*, and *MMP13* of OAC cells to Wnt-3A CM. **(A, B, and C)** OAC cells are treated with conditioned medium containing Wnt3A (Wnt3A-CM, 25%) for 48 hours. Expression levels of *AXIN2* **(A)**, *SOX9* **(B)**, and *MMP13* **(C)** mRNA are normalized to that without treatment. The mean and SD in three wells in a single experiment are indicated. *p < 0.05 and ***p < 0.005 by Student *t*-test.
(TIF)

S2 Fig Expressions of TNF and IL6 are not increased by DMM surgery in a rat model. **(A, B)** Three rats in each group had DMM and sham surgeries in the right and left knees, respectively, as described in Fig 6. Representative staining of knee joints with anti-TNF or anti-IL6 antibodies. Scale bar = 200 μm. Both cytokines are slightly expressed on the surface of cartilages but the levels are not changed by DMM surgery and fluoxetine treatment. **(C)** Human OAC cells are treated with or without fluoxetine (10 μM) in the presence of 10 mM LiCl for 48 hours. Expression levels of each mRNA are normalized to that without treatment. The mean and SD (*n* = 2 patients) are indicated.
(TIF)

S3 Fig Effects of fluoxetine, lithium, and mouse Wnt3A protein in ATDC5 cells. **(A, B)** ATDC5 cells are treated with indicated concentrations of fluoxetine and LiCl for 48 hours. Cell growth is quantified using the MTS assay. Data are presented as the mean and SD of 490-nm absorbance normalized by that without fluoxetine (*n* = 6 wells in an experiment). Fluoxetine shows slight toxicity at 25 and 50 μM. (**A)** *P*-value by one-way ANOVA of each condition is <0.0001. **(B)** *P*-value by Student’s *t*-test is 0.42. **(C, D)** Differentiated ATDC5 cells are treated with mouse Wnt3A proteins (mWnt3A; 100 ng/ml in 0.1% BSA) **(C)**, 10 mM NaCl **(D)**, or 10 mM LiCl **(D)** for 48 hours. Expression levels of each mRNA are normalized to that without treatment. The mean and SD in two wells each in two independent experiments (*n* = 4) are indicated. **(C)** *P*-values by one-way ANOVA of *Axin2*, *Sox9*, and *Mmp13* expressions are 0.011, 0.010, and 0.024, respectively. **(D)** *P*-values by one-way ANOVA of *Axin2*, *Sox9* and *Mmp13* expressions are 0.036, 0.028, and 0.018, respectively. *p < 0.05, **p < 0.01, and ***p < 0.005 by posthoc Tukey-Kramer test.
(TIF)

S4 Fig Fluoxetine downregulates LiCl-responsive *Matn1* in chondrogenically differentiated ATDC5 cells. ATDC5 cells are treated with ITS to induce chondrogenic differentiation. Differentiated ATDC5 cells are treated with 10 mM LiCl **(A)** or 10 μM Akt inhibitor **(B-D)** with or without 10 μM fluoxetine for 48 hours. Expression levels of each mRNA are normalized to that without treatment. The mean value of three wells in a single experiment is calculated first. Then, the means of three wells in three independent experiments are used to calculate the mean and SD (*n* = 3). *P*-values by one-way ANOVA are 0.016 **(A)**, 0.32 **(B)**, 0.089 **(C)**, 0.066 **(D)**, respectively. *p < 0.05 by posthoc Tukey-Kramer test.
(TIF)

S5 Fig A high dose of fluoxetine has no effects on OA progression or rather induces meniscus injury in a rat model. Three rats in each group have DMM and sham surgeries in the right and left knees, respectively. **(A)** Representative staining of knee joints with Safranin O and Fast-green. Scale bar = 400 μm. **(B)** OA progressions are evaluated by modified Mankin score at 8 weeks after the surgery. The mean and SD (*n* = 3 rats for each) are indicated. *P*-values by one-way ANOVA of each condition (DMM surgery and Sham surgery) are 0.035 and 0.42, respectively. *p < 0.05 by posthoc Tukey-Kramer test. The data suggest 200 μM fluoxetine rather worsens OA pathology.
(TIF)

S6 Fig Modified Mankin score at the lateral compartment and larger areas of stained knee joint sections shown in Fig 6. Sections crossing the narrowest interarticular space at the lateral compartment comprised of the lateral femoral condyle and the lateral tibial plateau are evaluated by modified Mankin score in three sham-operated knees and four DMM-operated knees. The modified Mankin scores at the lateral compartments are lower than those at the medial compartments indicated in Fig 6B. **(B)** Larger areas of stained sections of knee joints shown in Fig 6A, 6C and 6F. Scale bar = 400 μm.
(TIF)

S1 Table Primer sequences and efficiencies in qRT-PCR. (DOCX)

S1 Text Supplementary materials and methods. (DOCX)
