# Supplementary figures and images for: Fluoxetine ameliorates cartilage degradation in osteoarthritis by inhibiting Wnt/β-catenin signaling
Source: PLoS One. 2017 Sep 19;12(9):e0184388. doi: 10.1371/journal.pone.0184388 (PMC5604944; doi:10.1371/journal.pone.0184388)

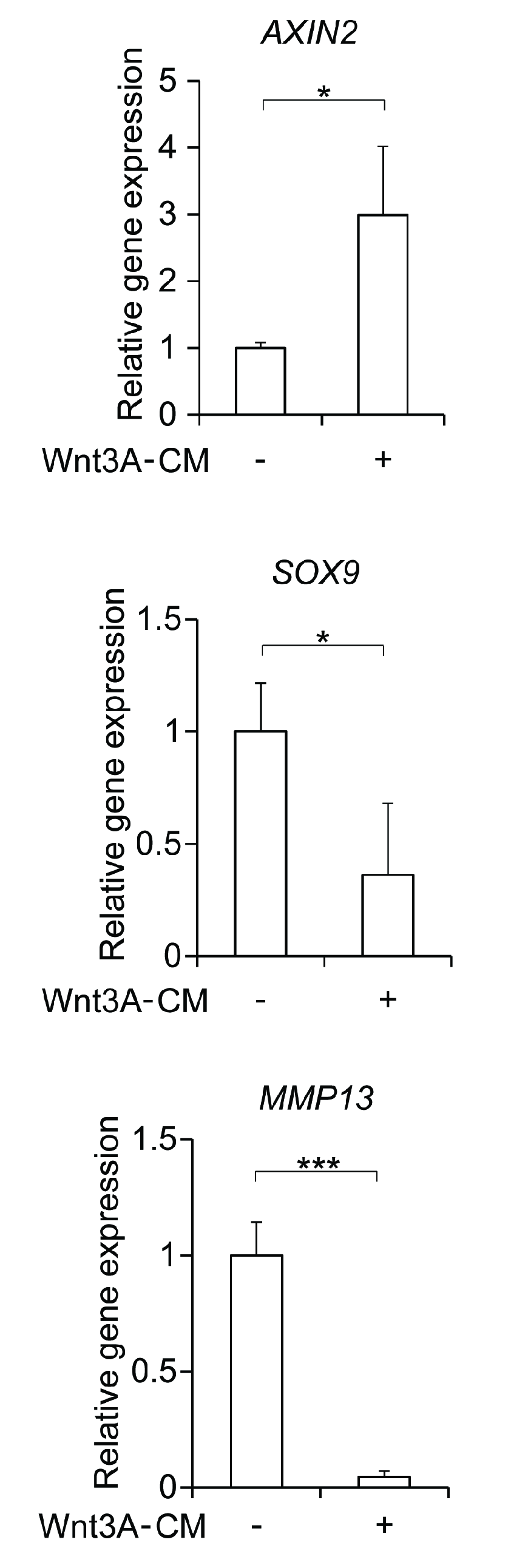

Supplement: S1 Fig — (A, B, and C) OAC cells are treated with conditioned medium containing Wnt3A (Wnt3A-CM, 25%) for 48 hours. Expression levels of AXIN2 (A), SOX9 (B), and MMP13 (C) mRNA are normalized to that without treatment. The mean and SD in three wells in a single experiment are indicated. *p < 0.05 and ***p < 0.005 by Student t-test. (TIF) [file pone.0184388.s001.tif]

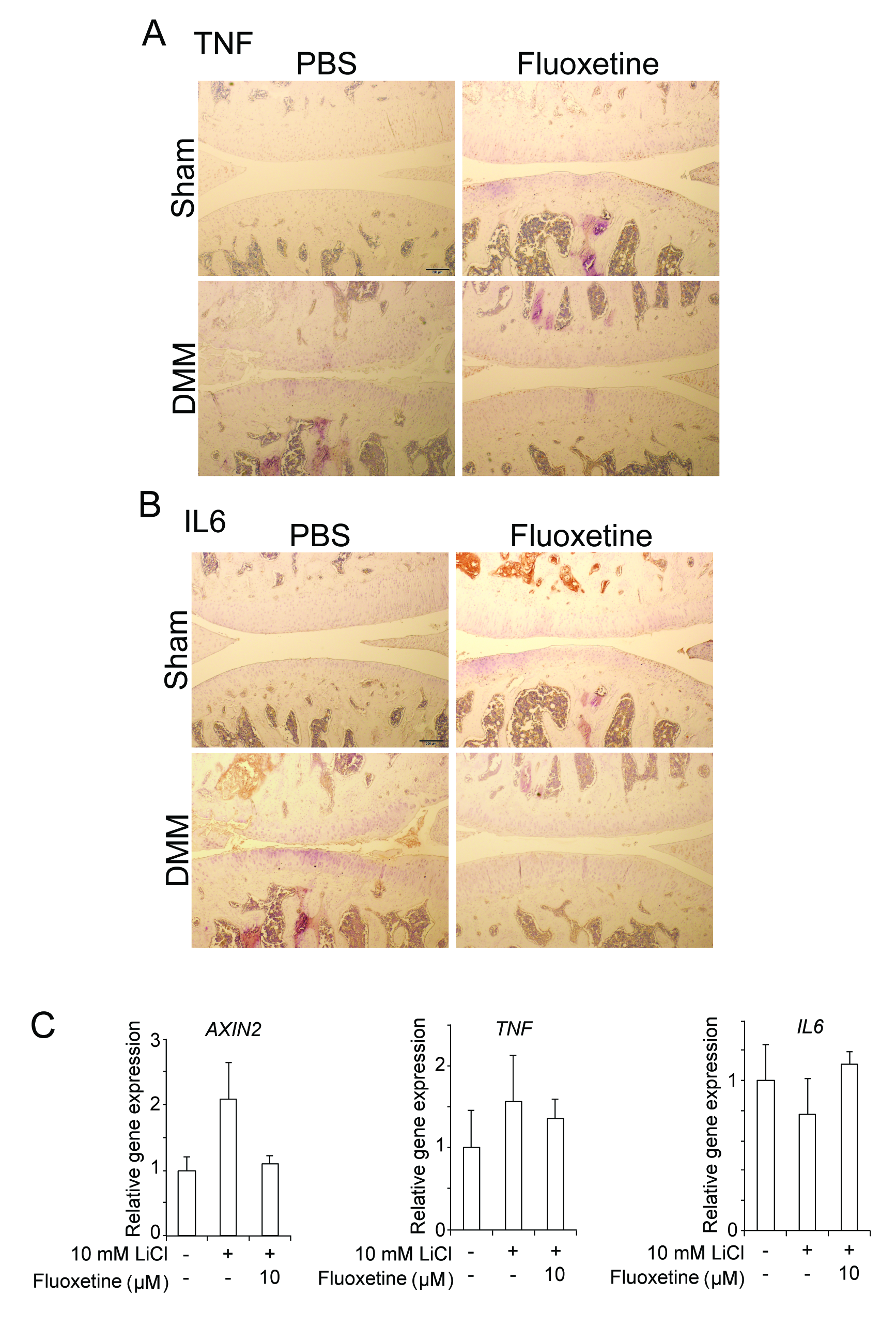

Supplement: S2 Fig — (A, B) Three rats in each group had DMM and sham surgeries in the right and left knees, respectively, as described in Fig 6. Representative staining of knee joints with anti-TNF or anti-IL6 antibodies. Scale bar = 200 μm. Both cytokines are slightly expressed on the surface of cartilages but the levels are not changed by DMM surgery and fluoxetine treatment. (C) Human OAC cells are treated with or without fluoxetine (10 μM) in the presence of 10 mM LiCl for 48 hours. Expression levels of each mRNA are normalized to that without treatment. The mean and SD (n = 2 patients) are indicated. (TIF) [file pone.0184388.s002.tif]

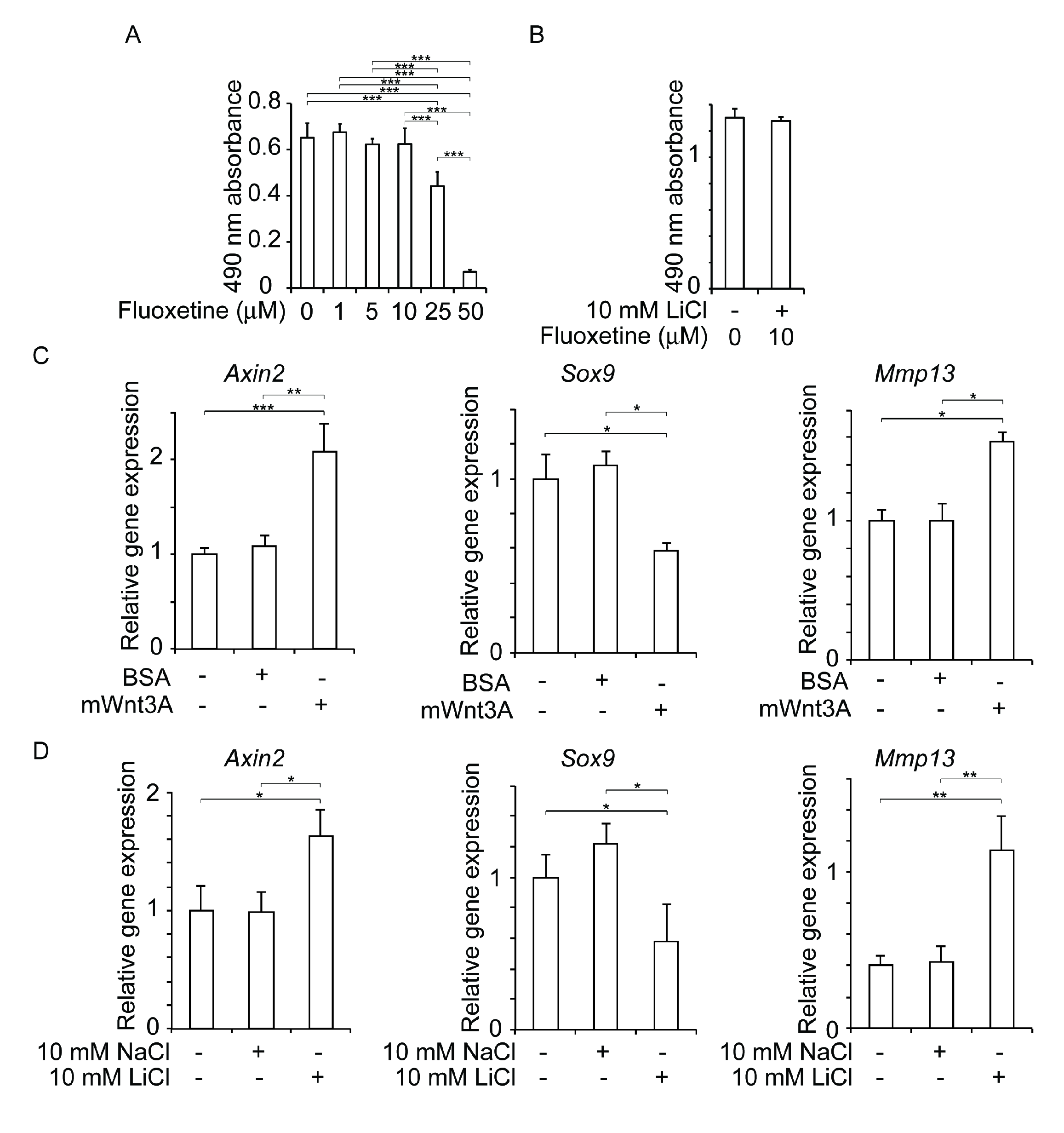

Supplement: S3 Fig — (A, B) ATDC5 cells are treated with indicated concentrations of fluoxetine and LiCl for 48 hours. Cell growth is quantified using the MTS assay. Data are presented as the mean and SD of 490-nm absorbance normalized by that without fluoxetine (n = 6 wells in an experiment). Fluoxetine shows slight toxicity at 25 and 50 μM. (A) P-value by one-way ANOVA of each condition is <0.0001. (B) P-value by Student’s t-test is 0.42. (C, D) Differentiated ATDC5 cells are treated with mouse Wnt3A proteins (mWnt3A; 100 ng/ml in 0.1% BSA) (C), 10 mM NaCl (D), or 10 mM LiCl (D) for 48 hours. Expression levels of each mRNA are normalized to that without treatment. The mean and SD in two wells each in two independent experiments (n = 4) are indicated. (C) P-values by one-way ANOVA of Axin2, Sox9, and Mmp13 expressions are 0.011, 0.010, and 0.024, respectively. (D) P-values by one-way ANOVA of Axin2, Sox9 and Mmp13 expressions are 0.036, 0.028, and 0.018, respectively. *p < 0.05, **p < 0.01, and ***p < 0.005 by posthoc Tukey-Kramer test. (TIF) [file pone.0184388.s003.tif]

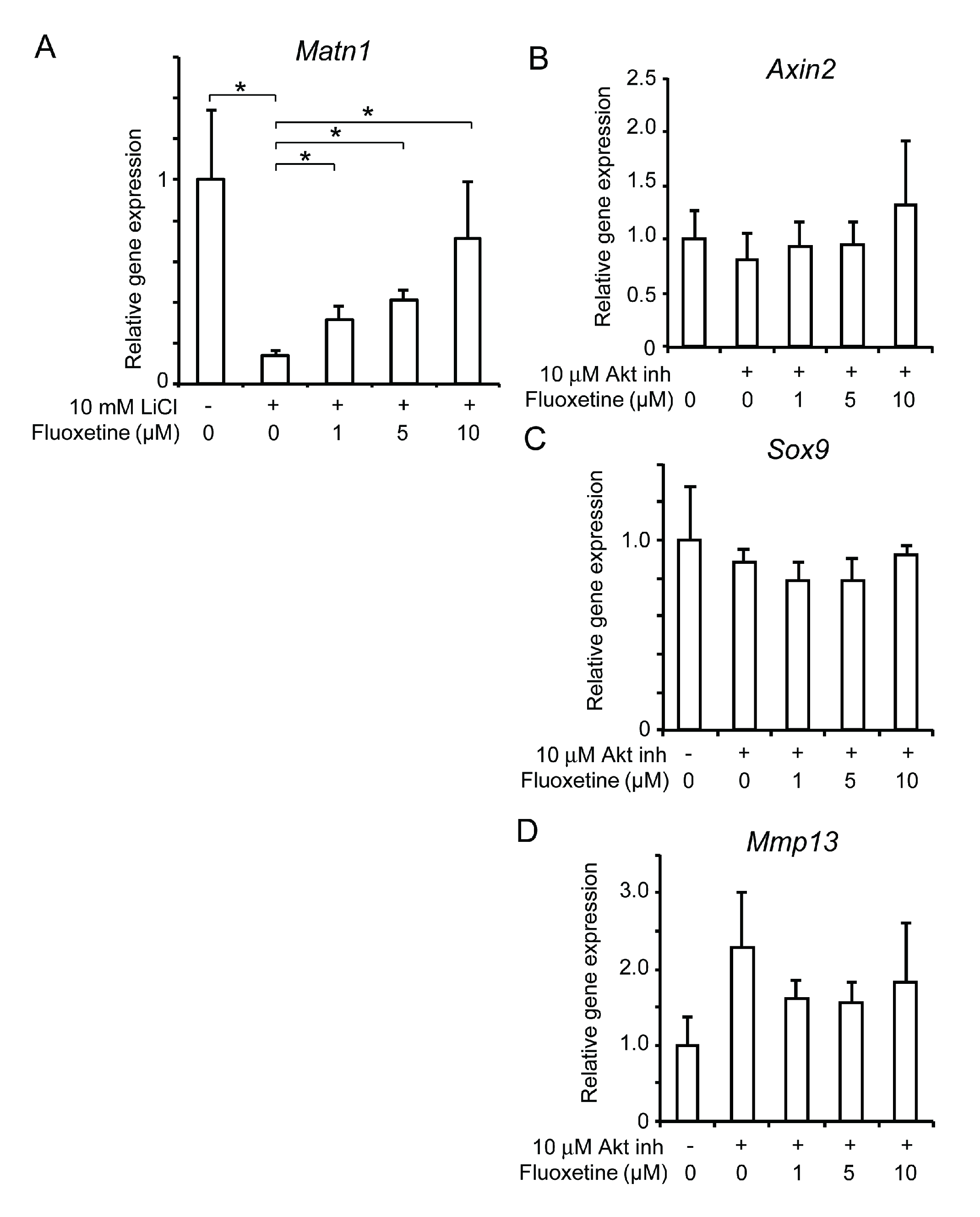

Supplement: S4 Fig — ATDC5 cells are treated with ITS to induce chondrogenic differentiation. Differentiated ATDC5 cells are treated with 10 mM LiCl (A) or 10 μM Akt inhibitor (B-D) with or without 10 μM fluoxetine for 48 hours. Expression levels of each mRNA are normalized to that without treatment. The mean value of three wells in a single experiment is calculated first. Then, the means of three wells in three independent experiments are used to calculate the mean and SD (n = 3). P-values by one-way ANOVA are 0.016 (A), 0.32 (B), 0.089 (C), 0.066 (D), respectively. *p < 0.05 by posthoc Tukey-Kramer test. (TIF) [file pone.0184388.s004.tif]

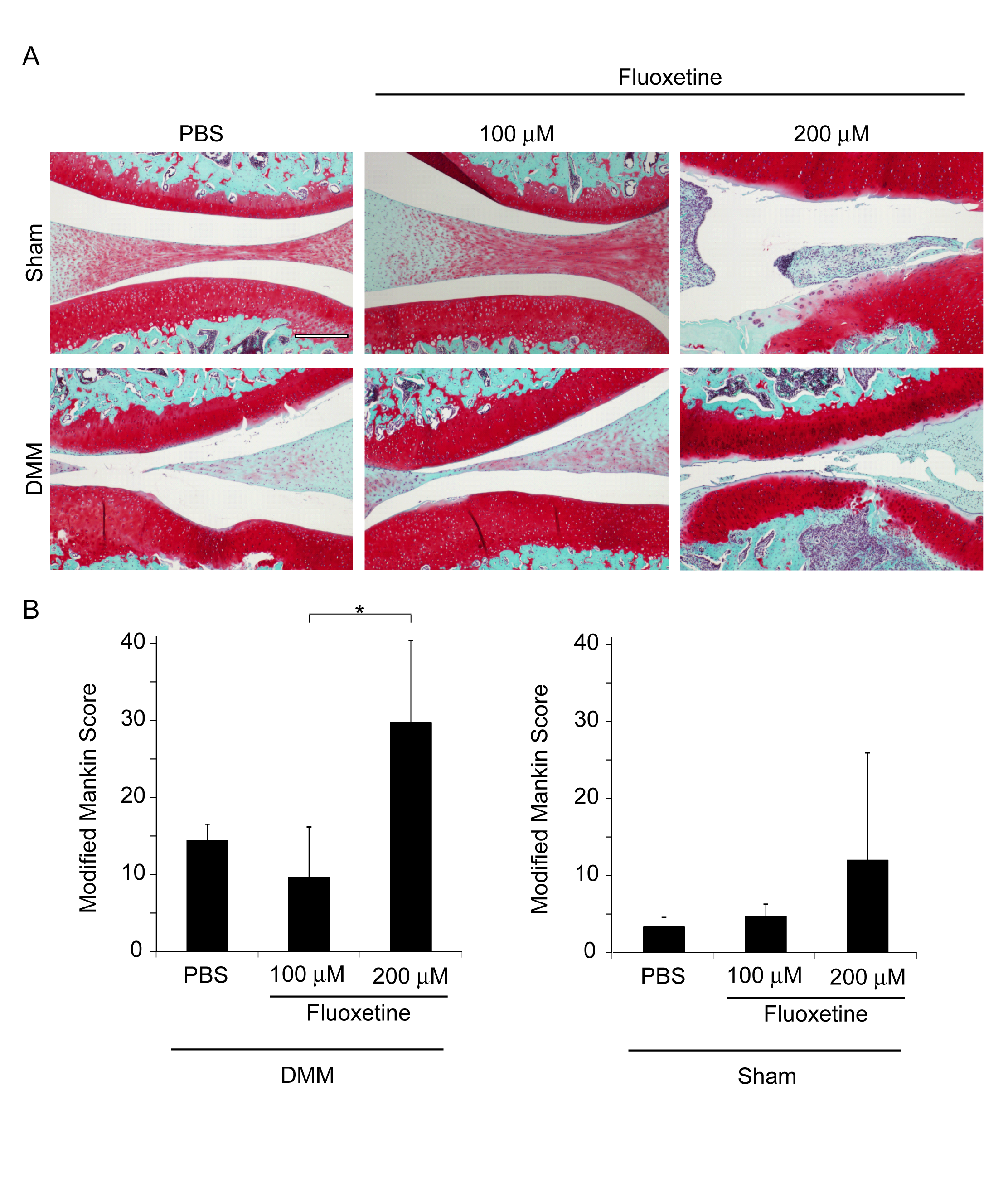

Supplement: S5 Fig — Three rats in each group have DMM and sham surgeries in the right and left knees, respectively. (A) Representative staining of knee joints with Safranin O and Fast-green. Scale bar = 400 μm. (B) OA progressions are evaluated by modified Mankin score at 8 weeks after the surgery. The mean and SD (n = 3 rats for each) are indicated. P-values by one-way ANOVA of each condition (DMM surgery and Sham surgery) are 0.035 and 0.42, respectively. *p < 0.05 by posthoc Tukey-Kramer test. The data suggest 200 μM fluoxetine rather worsens OA pathology. (TIF) [file pone.0184388.s005.tif]

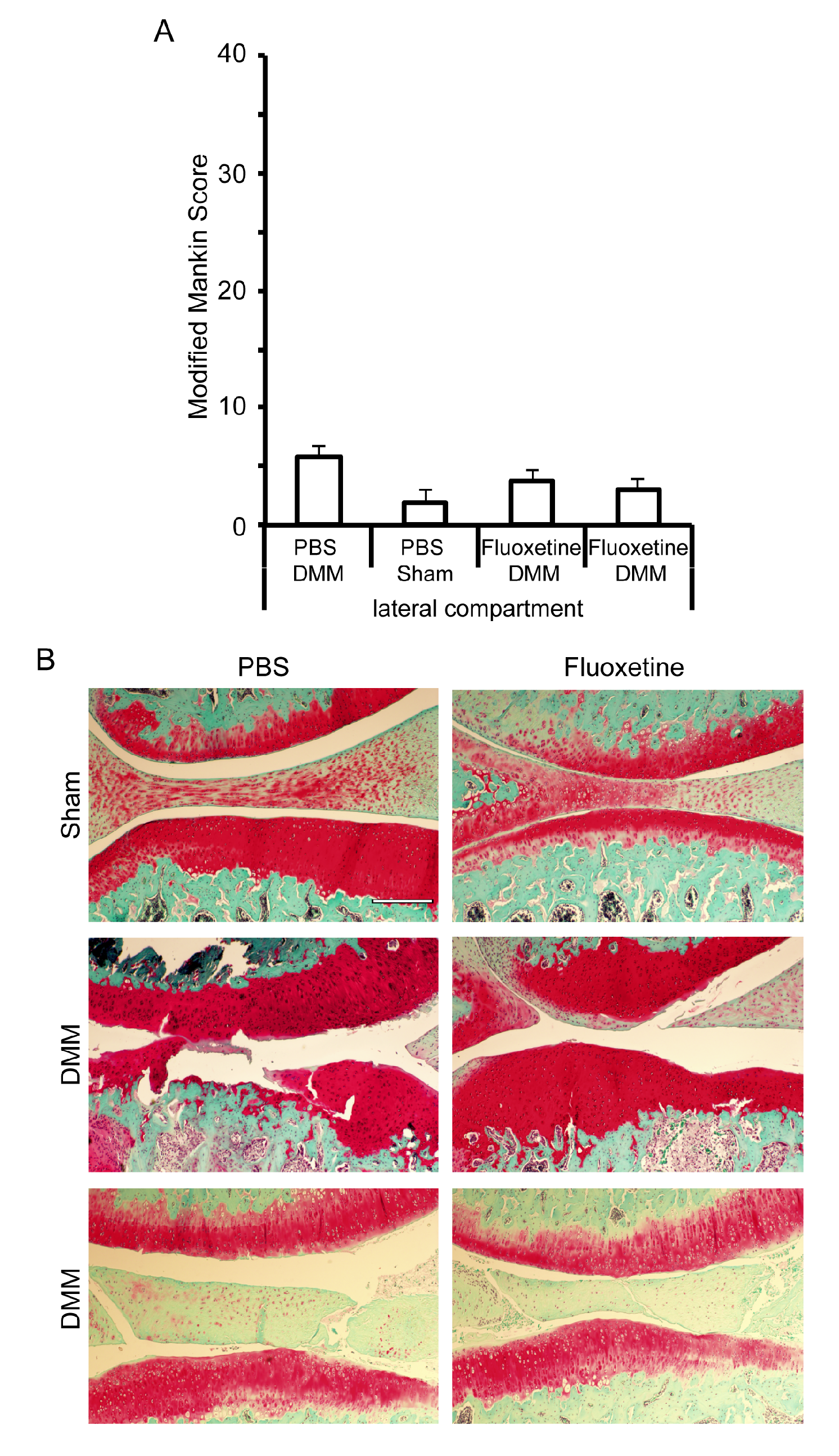

Supplement: S6 Fig — Sections crossing the narrowest interarticular space at the lateral compartment comprised of the lateral femoral condyle and the lateral tibial plateau are evaluated by modified Mankin score in three sham-operated knees and four DMM-operated knees. The modified Mankin scores at the lateral compartments are lower than those at the medial compartments indicated in Fig 6B. (B) Larger areas of stained sections of knee joints shown in Fig 6A, 6C and 6F. Scale bar = 400 μm. (TIF) [file pone.0184388.s006.tif]
