# Supplementary material for: Fluoxetine ameliorates cartilage degradation in osteoarthritis by inhibiting Wnt/β-catenin signaling
Source: PLoS One. 2017 Sep 19;12(9):e0184388. doi: 10.1371/journal.pone.0184388 (PMC5604944; doi:10.1371/journal.pone.0184388)
Supplement: S1 Table — (DOCX) [file pone.0184388.s007.docx]

**Supplementary Table 1. Primer sequences and efficiencies in qRT-PCR**

| Gene | Primer sequence | Efficiency |
| --- | --- | --- |
| Mouse *Sox9* | For. 5’ tatgtggatgtgtgcgtgtg 3’ | 2.22 |
|  | Rev. 5’ ccagccacagcagtgagtaa 3’ |  |
| Mouse *Mmp13* | For. 5’ agttgacaggctccgagaaa 3’ | 2.13 |
|  | Rev. 5’ ggcactccacatcttggttt 3’ |  |
| Mouse *Axin2* | For. 5’ ctccccaccttgaatgaaga 3’ | 2.05 |
|  | Rev. 5’ gtttccgtggacctcacact 3’ |  |
| Mouse *Matn1* | For. 5’ tccacagggaccatgacc 3’ | 1.98 |
|  | Rev. 5’ ctggccccttctgtgtca 3’ |  |
| Human *TNF* | For. 5’ cccgagtgacaagcctgtag 3’ | 1.83 |
|  | Rev. 5’ gatggcagagaggaggttgac 3’ |  |
| Human *IL6* | For. 5’ acagccactcacctcttcag 3’ | 1.91 |
|  | Rev. 5’ ccatctttttcagccatcttt 3’ |  |
| Human *SOX9* | For. 5’ cgaagaaagagaggaccaaccag 3’ | 1.99 |
|  | Rev. 5’ tcgctgctccatttagccaagg 3’ |  |
| Human *MMP13* | For. 5’ ggtggtgatgaagatgatt 3’ | 1.95 |
|  | Rev. 5’ tcagtcatggagcttgct 3’ |  |
| Human *AXIN2* | For. 5’ taccggaggatgctgaaggc 3’ | 1.94 |
|  | Rev. 5’ ccactggccgattcttcctt 3’ |  |

For., forward primer; Rev., reverse primer
