# Supplementary material for: Fluoxetine ameliorates cartilage degradation in osteoarthritis by inhibiting Wnt/β-catenin signaling
Source: PLoS One. 2017 Sep 19;12(9):e0184388. doi: 10.1371/journal.pone.0184388 (PMC5604944; doi:10.1371/journal.pone.0184388)
Supplement: S1 Text — (DOCX) [file pone.0184388.s008.docx]

**Supplementary Materials and Methods**

**Alcian blue staining**

Mouse chondrogenic progenitor ATDC5 cells (Riken BioResource Center, Japan) were cultured in a mixture of DMEM and Ham's F12 medium (DMEM/F12, Sigma) supplemented with 5% fetal bovine serum (FBS, Thermo Fisher Scientific). After culturing for 2 weeks with 1% insulin-transferrin-sodium selenite (ITS, Sigma) for chondrogenic differentiation, ATDC5 cells were treated with 0, 1, and 10 μM fluoxetine in the presence of 10 mM LiCl or 20% Wnt3A-CM for 48 hours. As we expected that fluoxetine was able to suppress moderately activated Wnt/β-catenin signaling, but to a less extent for strongly activated Wnt/β-catenin signaling, we used 20% Wnt3A-CM instead of 25% Wnt3A-CM that we used for drug screening. Cells were fixed with methanol for 30 minutes at −20°C and stained overnight with 0.5% Alcian Blue 8 GX (Sigma) in 1 N HCl. The cells were lysed in 200 µl of 6 M guanidine HCl for 6 hours at room temperature [1], and the optical density of the extracted dye was quantified at 650 nm using PowerScan4 (DS Parma Biomedical).

**Total RNA extraction and real-time RT-PCR analysis**

The differentiated ATDC5 cells were treated with 0, 1, 5, and 10 μM fluoxetine in the presence of 10 mM LiCl or 20% Wnt3A-CM for 48 hours. OAC cells were treated with 25% Wnt3A-CM. Total RNA was isolated using Trizol from these samples, and the first strand cDNA was synthesized with ReverTra Ace (Toyobo). We quantified mRNA expressions of *Axin2*, *Sox9*, *Mmp13*, *TNF,* and *IL6* using LightCycler 480 Real-Time PCR (Roche) and SYBR Green (Takara). mRNA levels were normalized for that of *Gapdh*. Primer sequences, which were previously reported [2-4] and synthesized by us, as well as the efficiencies in qRT-PCR, are shown in Supplementary Table 1.

**Western blotting**

To detect β-catenin accumulation, we inhibited protein degradation by an inhibitor of ubiquitin-conjugated proteins, MG-132 (Wako, 135-16253). Differentiated ATDC5 cells were cultured for 24 hours with 2 µM MG-132 and then treated with 2 µM MG-132 plus 0, 1, 5, and 10 μM fluoxetine in the presence of 10 mM LiCl for 48 hours. These samples were lysed in the ice-cold RIPA Lysis Buffer (Santa Cruz) supplemented with the proteinase and phosphatase inhibitors (PhosSTOP, Sigma). Whole cell lysates were separated on SDS-PAGE and transferred to a nitrocellulose membrane. For preparation of the nuclear and cytoplasmic fractions from human OAC cells, the cells were cultured for 48 hours with LiCl with or without fluoxetine, washed with PBS and resuspended in buffer A containing 10 mM HEPES-KOH (pH 7.8), 10 mM KCl, 0.1 mM EDTA and 0.1% NP-40, 10 mM sodium pyrophosphate, 1 µg/µl aprotinin, 1 µg/µl leupeptin, 1 µg/µl pepstatin A, 1 mM PMSF, 1 mM sodium orthovanadate, and the Phosphatase Inhibitor Cocktail (Sigma). The cells were then vortexed and centrifuged at 5,000 x *g* for 2 min. The supernatant was collected as a cytoplasmic fraction. The pellet was washed with buffer A and resuspended in buffer C containing 50 mM HEPES-KOH (pH 7.8), 420 mM KCl, 0.1 mM EDTA, 5 mM MgCl_2_, 2% glycerol, 10 mM sodium pyrophosphate, 1 µg/µl aprotinin, 1 µg/µl leupeptin, 1 µg/µl pepstatin A, 1 mM PMSF, 1 mM sodium orthovanadate, and the Phosphatase Inhibitor Cocktail. The pellet was vortexed and incubated at 4°C on a rotating shaker for 30 min. The pellet was then centrifuged at 20,600 x *g* at 4ºC for 15 min. The supernatant was collected as a nuclear fraction. The expression levels of β-catenin and phosphorylated β-catenin protein were determined by Western blotting using antibodies against β-catenin (dilution 1:1000, BD Transduction Laboratories, 610154), phospho-β-catenin at Ser33/37/Thr41 (dilution 1:2000, CST, #9561), and β-actin (dilution 1:200, Santa Cruz, sc47778). Three independent experiments were performed, and signal intensities were quantified using ImageJ software.

**Co-immunoprecipitation assay for Axin1**

To detect β-catenin accumulation, MG-132 was used to inhibit protein degradation by proteasome. Mouse fibroblast L cells have higher protein expression levels of GSK3, CK1, and Axin1 than HCS-2/8 and ATDC5 cells [5], and were suitable for co-immunoprecipitation of Axin1 and β-catenin. L cells were cultured for 48 hours with 2 µM MG-132 and then lysed in the ice-cold RIPA Lysis Buffer (Santa Cruz) supplemented with the proteinase inhibitors (Roche). The lysate was centrifuged and the supernatant was added with or without fluoxetine in 0.1% DMSO. Using the supernatant, Axin1 protein was immunoprecipitated using 1 µg anti-Axin1 antibody (Millipore, 05-1579) or normal mouse IgG (Santa Cruz, sc-2025) attached to protein G Sepharose beads (GE Life Science). Immunoprecipitated molecules were detected by Western blotting as described above using antibodies against β-catenin (dilution 1:1000, BD Transduction Laboratories, 610154), GSK3α/β (dilution1:500, Santa Cruz, sc7291), and CK1ε (dilution 1:200, Santa Cruz, c-20, sc6471).

**Immunofluorescence staining with OAC cells**

OAC cells were treated with or without 10 μM fluoxetine in the presence of 10 mM LiCl. After 48 hours, cells were fixed with 4% paraformaldehyde at room temperature. The specimens were then treated with a blocking buffer including 2% goat serum in 0.5% Triton-X100 for 60 minutes and incubated with mouse anti-β-catenin antibody (dilution 1:500, BD Transduction Laboratories, 610153) at 4°C overnight. The specimens were incubated with Alexa 488-conjugated goat anti-mouse IgG secondary antibody (dilution 1:500, Thermo Fisher Scientific, A-11001) at room temperature for 1 hour. Finally, the specimens were mounted in VectaShield containing 1.5 µg/ml diamidino-2-phenylindole (DAPI, Vector Laboratories) and visualized using FSX100 (Olympus). The average signal intensities for β-catenin normalized to the number of DAPI positive-cells were blindly quantified using MetaMorph (Molecular Device). Briefly, signals more than 4 µm in diameter were automatically taken as positive signals. The number of cells was calculated by counting the number of DAPI-positive areas. Signals of β-catenin within the DAPI-positive areas were used to calculate the signal intensities of nuclear β-catenin. Cells with similar average intensities of β-catenin signals in both nuclear and cytoplasmic regions were counted as β-catenin positive cells.

**MTS assay**

ATDC5 cells (5 × 10^3^ cells per well) were seeded in a 96-well culture plate and incubated for 48 hours in the presence of fluoxetine and/or LiCl. Cell proliferation was quantified by the MTS assay (Cell 96 AQueus One Solution Cell Proliferation Assay, Promega) according to the manufacturer’s instructions.

**References**

1. De Bari C, Dell'Accio F, Luyten FP. Human periosteum-derived cells maintain phenotypic stability and chondrogenic potential throughout expansion regardless of donor age. Arthritis Rheum. 2001;44(1):85-95. Epub 2001/02/24. doi: 10.1002/1529-0131(200101)44:1<85::AID-ANR12>3.0.CO;2-6. PubMed PMID: 11212180.

2. Tian J, Yan J, Wang W, Zhong N, Tian L, Sun J, et al. T-2 toxin enhances catabolic activity of hypertrophic chondrocytes through ROS-NF-kappaB-HIF-2alpha pathway. Toxicol In Vitro. 2012;26(7):1106-13. doi: 10.1016/j.tiv.2012.07.002. PubMed PMID: 22800716.

3. Nalesso G, Sherwood J, Bertrand J, Pap T, Ramachandran M, De Bari C, et al. WNT-3A modulates articular chondrocyte phenotype by activating both canonical and noncanonical pathways. J Cell Biol. 2011;193(3):551-64. doi: 10.1083/jcb.201011051. PubMed PMID: 21536751; PubMed Central PMCID: PMCPMC3087013.

4. Salemi S, Rethage J, Wollina U, Michel BA, Gay RE, Gay S, et al. Detection of interleukin 1beta (IL-1beta), IL-6, and tumor necrosis factor-alpha in skin of patients with fibromyalgia. J Rheumatol. 2003;30(1):146-50. PubMed PMID: 12508404.

5. Lin Y, Ohkawara B, Ito M, Misawa N, Miyamoto K, Takegami Y, et al. Molecular hydrogen suppresses activated Wnt/beta-catenin signaling. Sci Rep. 2016;6:31986. doi: 10.1038/srep31986. PubMed PMID: 27558955; PubMed Central PMCID: PMCPMC5001535.
